# Divide and conquer: A perspective on biochips for single-cell and rare-molecule analysis by next-generation sequencing

**DOI:** 10.1063/1.5095962

**Published:** 2019-06-25

**Authors:** A. C. Lee, Y. Lee, D. Lee, S. Kwon

**Affiliations:** 1 Interdisciplinary Program in Bioengineering, Seoul National University, Seoul 08826, South Korea; 2 Department of Electrical and Computer Engineering, Seoul National University, Seoul 08826, South Korea; 3 BK21+ Creative Research Engineer Development for IT, Seoul National University, Seoul 08826, South Korea; 4 Institutes of Entrepreneurial BioConvergence, Seoul National University, Seoul 08826, South Korea; 5 Seoul National University Hospital Biomedical Research Institute, Seoul National University Hospital, Seoul 03080, South Korea

## Abstract

Recent advances in biochip technologies that connect next-generation sequencing (NGS) to real-world problems have facilitated breakthroughs in science and medicine. Because biochip technologies are themselves used in sequencing technologies, the main strengths of biochips lie in their scalability and throughput. Through the advantages of biochips, NGS has facilitated groundbreaking scientific discoveries and technical breakthroughs in medicine. However, all current NGS platforms require nucleic acids to be prepared in a certain range of concentrations, making it difficult to analyze biological systems of interest. In particular, many of the most interesting questions in biology and medicine, including single-cell and rare-molecule analysis, require strategic preparation of biological samples in order to be answered. Answering these questions is important because each cell is different and exists in a complex biological system. Therefore, biochip platforms for single-cell or rare-molecule analyses by NGS, which allow convenient preparation of nucleic acids from biological systems, have been developed. Utilizing the advantages of miniaturizing reaction volumes of biological samples, biochip technologies have been applied to diverse fields, from single-cell analysis to liquid biopsy. From this perspective, here, we first review current state-of-the-art biochip technologies, divided into two broad categories: microfluidic- and micromanipulation-based methods. Then, we provide insights into how future biochip systems will aid some of the most important biological and medical applications that require NGS. Based on current and future biochip technologies, we envision that NGS will come ever closer to solving more real-world scientific and medical problems.

## I. BIOCHIPS WILL PLAY A PIVOTAL ROLE IN APPLICATIONS BASED ON NGS

**FIG. 1.**
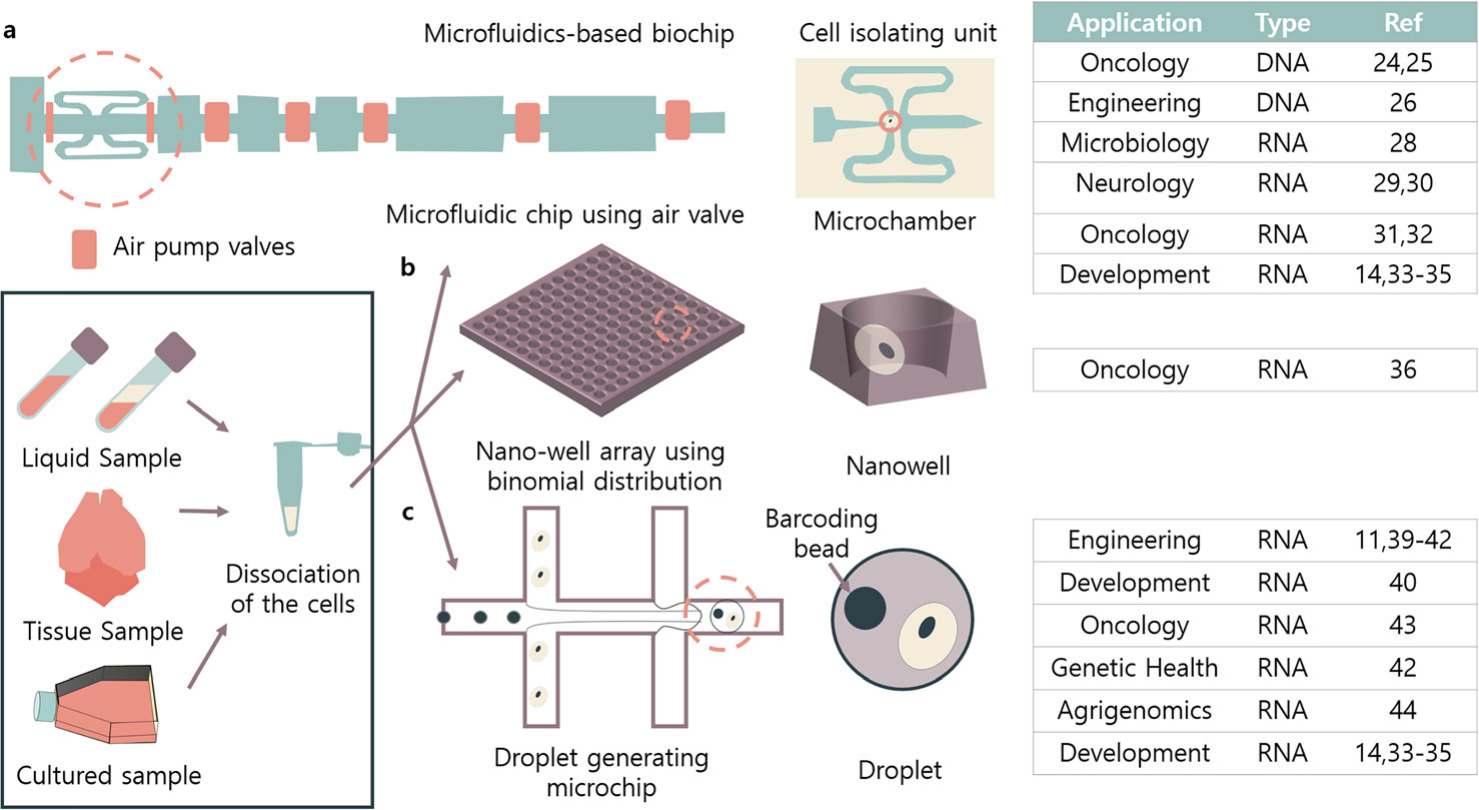
Representative microfluidics-based biochips and representative references. Microfluidics-based biochips can be categorized into three major platforms: (a) microfluidic channel-based, (b) well-array-based, and (c) droplet-based. After biological samples are dissociated into solution, the samples are processed through these biochips and can be applied to various fields in biology and medicine.

**FIG. 2.**
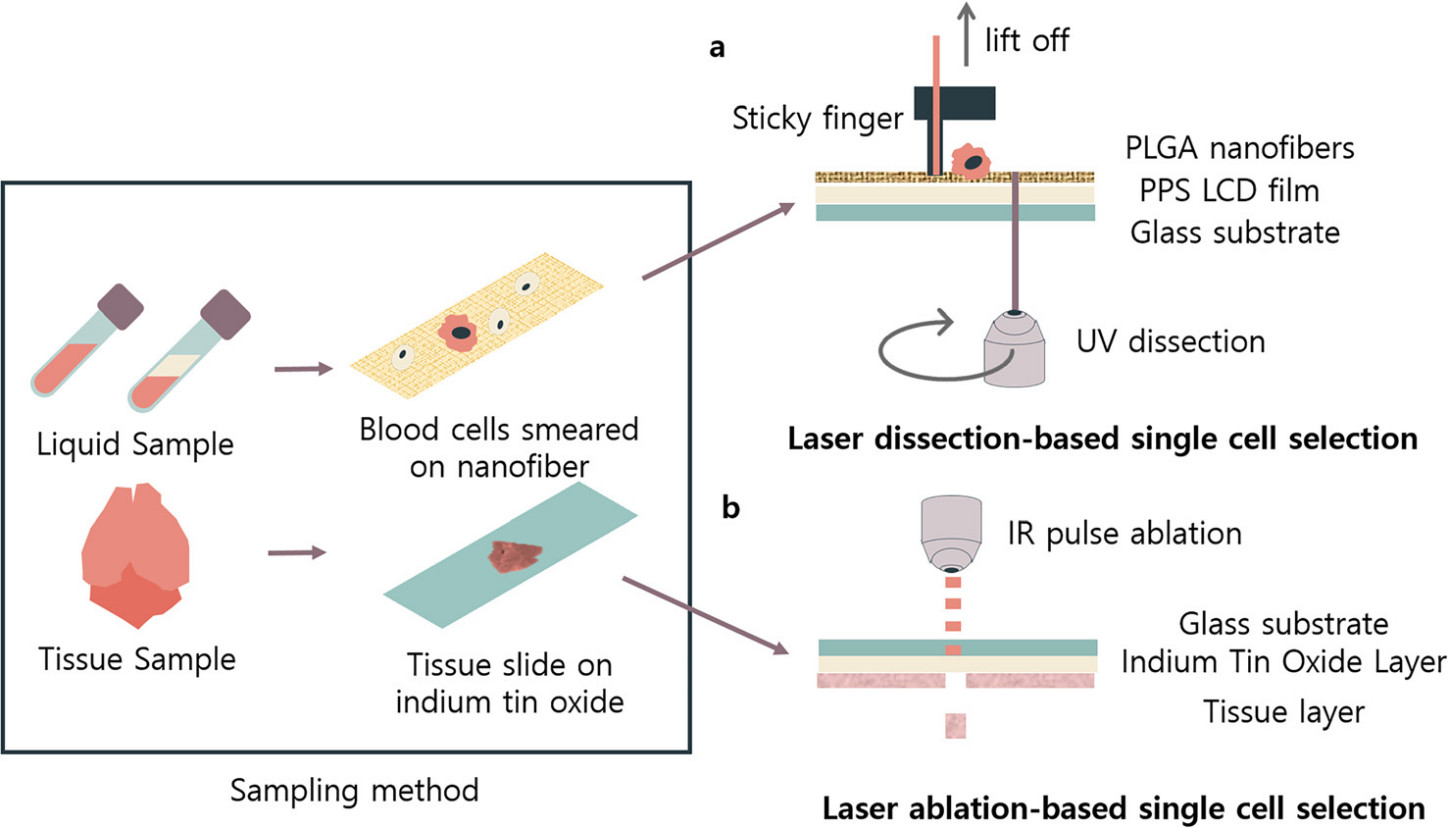
Representative laser-based biochips. Laser-based biochips enable a connection between the micro- and macroworlds by transferring analytes of interest to conventional tubes that are easy-to-handle. Two types are demonstrated: (a) laser dissection-based and (b) laser ablation-based single-cell selection methods. Also, laser-based methodologies do not require the samples to be dissociated into solution, leaving room for the analysis of sample images.

**FIG. 3.**
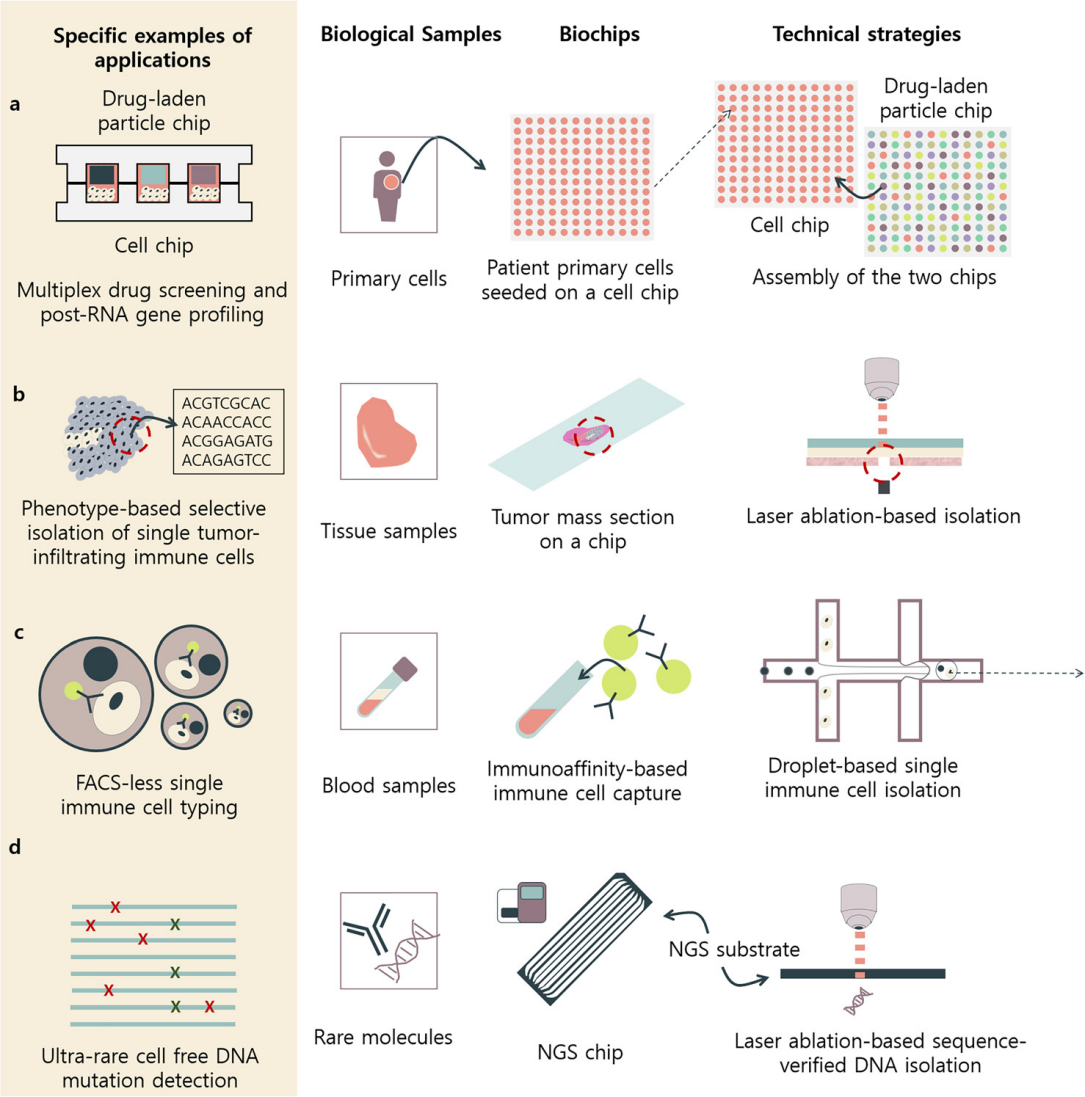
Perspectives on potential biochips used for next generation sequencing for promising applications in biotechnology. Specific examples of biological and medical applications that the future biochips can aid to develop are demonstrated. The examples are conceptualized based on the previous literature reported for other purposes but have significant potential for NGS-related applications. These biochips will aid in better understanding and solving unmet needs in diverse biological and clinical fields. (a) Multiplex high-throughput drug-screening and post-RNA profiling will allow not only clinical genetic testing according to drug applications but also scientific investigations on how molecules affect certain biological cells. (b) Phenotype based cell isolation using biochip technologies will allow selective enrichment of biological cells of interest for deep sequencing. (c) Because droplet-based single-cell isolation utilizes FACS for pre-enrichment of the cells of interest, surface-protein based capture of cells integrated with droplet-based single-cell isolation methods could be a powerful tool in analyzing heterogeneous populations of immune cells. (d) Some of the most important biomarkers are ultrarare. These can be simply enriched when using NGS chip based auxiliary technologies.

Next-generation sequencing (NGS) technologies that have emerged from advances in optics, biochemistry, information, and engineering technologies, such as semiconductors, can deliver more rapid, inexpensive, and accurate genetic information than conventional Sanger sequencing. In particular, biochip technologies are crucial in advancing NGS, as shown by several platforms that are currently commercially available.^1,2^ Through this convergence of technologies, NGS has revolutionized clinical genomics, direct-to-consumer genetic screening, metagenomics, pharmacogenetics, neuroscience, and stem cells.^1–7^ Similarly, the initial application of NGS was focused on scientific discoveries achieved by sequencing DNA or RNA in bulk.^8^ Although bulk sequencing is widely used for important applications,^9–11^ it is inadequate for some emerging applications from which the genetic molecules of importance exist in small amounts among a large pool of molecules. Some specific examples include cellular heterogeneity,^10,12,13^ rare circulating tumor cells (CTCs),^14,15^ single cells atlasing in brain or immune cells,^16,17^ and circulating tumor DNA (ctDNA). Such examples represent only a few of the biological samples that are difficult to analyze using bulk sequencing. However, NGS platforms cannot separate the bulk data at the single-cell or molecule level unless they are processed in advance and molecularly labeled. If rare molecules are sequenced within the larger pool, a higher sequencing depth is required to collect data for the molecule of interest. However, if the raw sample of mixed molecules can be preprocessed and rare DNA molecules can be enriched and labeled separately, the read signals of importance will be recovered. Therefore, the upcoming trend is improving the resolution of NGS, and most of the solutions for improvement are the result of performing specific preprocessing before sequencing. While NGS has already spawned a series of discoveries and biotechnologies, the potential for this technology to continue to fuel future discoveries has catalyzed the development of numerous biochip technologies, in particular, for compartmentalizing single cells and molecules at the micro- or nanoscale. When compartmentalized at the micro- or nanoscale using a biochip, single cells or molecules can be separately labeled at a high resolution. This high-resolution signal enables not only a thorough investigation of complex and heterogeneous biology but also the practical implementation of NGS in medicine. Furthermore, the reduced consumption of materials and reagents saves preparation costs. Other advantages of biochip technologies include biocompatibility, flexibility, simplicity in design, and reliability. Therefore, microchips for processing raw samples based on these advantages have been developed because all current NGS technologies require the DNA to be sequenced at a certain length and concentration. In that sense, biochips have played and will continue to play a pivotal role in biotechnologies through improving NGS technologies. We herein describe several previously reported biochips for preprocessing samples for applications such as single-cell analysis or liquid biopsy. We first describe two different approaches and applications categorized as (1) microfluidic-based single-cell separation (Fig. 1) and (2) laser-based single-cell separation in biochips (Fig. 2). Next, we discuss future applications and upcoming fields where biochip technologies can be applied for NGS (Fig. 3).

## II. PIONEERING SINGLE-CELL ISOLATION FOR NGS: MICROFLUIDIC-BASED BIOCHIPS

Single-cell isolation is important because all biological samples are composed of heterogeneous populations of cells, and each cell is different. For example, cancer cells accumulate nucleotide variations or copy number variations to evolve into a heterogeneous tumor mass.^12,18^ Therefore, if cancer cells are dissociated in bulk and sequenced together, rare cancer cells' genotypic information, such as nucleotide or copy number variations, will be ignored due to the high signal of the bulk. To enable the above analysis, it was natural that a microfluidic-based biochip was developed because it is well matched to the size of a single cell and has characteristics that allow it to easily integrate with existing bulk biochemistry. A variety of microfluidic single-cell separation chips have been developed, ranging from fluorescence activated cell sorting (FACS), which can separate single cells by fluorescence-based methods, to Fluidigm, which is the first commercialized single-cell chip [Fig. 1(a)]. However, these chips were limited in terms of throughput. To overcome these limitations, droplet-based technologies were developed, but these have some disadvantages such as loss of spatial information within bulk samples. In recent years, it has become possible to perform a cell-atlasing with a single-cell experiment of up to 1000 units.

### A. From traditional methods to high-throughput technologies: Microfluidic technologies with solid-phase compartments

Fluorescence activated cell sorting (FACS) is perhaps the gold standard for the mechanized separation of single cells.^19^ Contemporary FACS methods utilize a laser source that is applied to fluorescently labeled cells that pass through the device. The cells are sorted based on the light scatter and fluorescence emission signals.^20^ FACS machines have been developed, which achieve a higher throughput and automate the process via addition of fluorescent dye labels to the cells. Although many commercial instruments based on FACS are widely used, these instruments tend to be bulky and expensive and therefore are often difficult to implement in many applications, even though the throughput is high. Therefore, a miniaturized microfluidic device for FACS has been developed.^21^ The microfluidic device is composed of InGaN LEDs (light emitting diodes) and laser diodes that can replace bulky gas lasers, highly efficient avalanche photodiodes with selective optical filter coatings that serve as photo multiplier tubes, and a substrate with microfluidic channels and reservoirs that serve as the path of the cells to be analyzed. Still, limitations in fluorescence types have restricted this method to sorting only 30 types of cells, which is far lower than the number of cell types in many other applications. Furthermore, this method relies on only cell surface markers for analysis.

The SMARTer ICELL8 multisample nanodispensor from Takara (previously from Wafergen) uses a biochip with small wells where cells can be dispensed into the chip [Fig. 1(b)]. There are 5184 nanowells for specific concentrations, and approximately 1000 to 1800 single cells are isolated in the 5184 nanowells according to a Poisson distribution. Subsequently, the RNA is captured by probes, the probe is labeled with unique barcodes that mark their original positions, and the captured RNAs are then pooled into a solution for further processing for NGS, according to the demand of the users. The core strength of the nanodispensor is its high throughput and ability to acquire RNA from 1000 single cells simultaneously and the low quantity of buffer that can be used for bio-chemistry thanks to the nanosized well. An example includes the investigation of phenotypic diversity in breast cancer.^22^ Although the biochip from a SMARTer ICELL8 multisample nanodispensor system allows for a higher throughput than that of a FACS system, the ICELL8 system is not yet available for DNA sequencing because a technique for molecularly barcoding the whole genome has not yet been reported. If the DNA of single cells can be barcoded and sequenced for meaningful outputs such as the copy number or single nucleotide variants, the ICELL8 system may be utilized for DNA sequencing.

Biochips for the C1 platform from Fluidigm utilize microfluidic valves and pumps developed by Stephen Quake's group [Fig. 1(a)]. The primary function of these biochips is to trap single cells in the entrance of the small channel; the cells are then lysed, and the DNA or RNA within is amplified and harvested in the subsequent small chamber controlled by microfluidic valves and pumps. The elaborate state-of-the-art integrated fluidic circuit (IFC) chip enables automated sequencing preparation in nanoliter volumes.^23^ Another strength of this platform is that the application is not limited to DNA or RNA sequencing because the IFCs enable a complex and creative manipulation of the molecules inside single cells. For example, the multiple displacement amplification method can be used and random hexamers primed throughout the genome; then, phi29 polymerase amplifies the DNA by hyper-branching.^24–26^ Another important feature of this microfluidic platform is that the captured cells can be imaged using a fluorescence microscope to identify the cells. This platform has been used in fields including microbiology,^27^ neurology,^28,29^ oncology,^30,31^ and stem cells.^13,32–34^

Although these widely applied microfluidics-based platforms are automated and yield high throughputs, challenges still exist. For example, the so-called “doublet problem,” or the harvest of two cells in a single sample, is primarily observed in C1 (11%–44%).^35^ However, this problem was accounted for with the imaging module of C1 because the on-chip capture in both the C1 and ICELL8 systems allows for imaging and matching. If the sample was a doublet, it could be identified and excluded from downstream analysis. Another example is the effect of dissociation. All cells must be dissociated into solution before they are input to the biochips, thus losing or modifying their genuine phenotypic information. Although the biochip platforms allow for imaging before analysis, this step only allows for the verification of whether the sample was a doublet or stained with the desired molecules, i.e., immunofluorescently stained. Therefore, the broader context provided by the tissue and microenvironment, where the cells originally existed, is lost during the process.

### B. Droplet-based single-cell isolation technologies: Microfluidic technologies with liquid-phase compartments

Single-cell isolation also becomes important in single-cell atlasing in the brain, because the brain is composed of neurons, astrocytes, Schwann cells, and other types of cells, all of which express genes to different levels, even for cells of the same type. Thus, analyzing single cells increases our understanding of how the orchestration of different cells occurs. In other words, rather than placing the orchestrated population of cells onto NGS platforms, separating individual cells and matching barcodes that can map each nucleic acid molecule to the single cells they originated from enables a deeper analysis of the individual read signals. To enable the above analyses, droplet-based separation methods utilize tiny aqueous droplets in oil as the compartments, enabled by microfluidic devices that precisely combine aqueous and oil flows.^36,37^ Such methods have emerged to yield the simultaneous throughput of thousands of single cells, large enough to better cover specific organs or tumor masses than microfluidics-based biochips.^10^

Macosko *et al.* reported the highly parallel RNA sequencing of single cells in nanoliter droplets^10^ [Fig. 1(c)]. Inside the droplets are microparticles that yield single-cell transcriptomes when the cell is lysed inside the droplet. The same principle used for microparticle labeling is applied to hydrogels.^38^ In their paper, Kirschner's group reported single embryonic stem-cell transcriptomics to reveal the heterogeneity in subpopulations of embryonic stem cells. A slight variation of the technique, namely drop-seq, involves the incorporation of photocleavable spacers between the hydrogels and molecular baits, such that the baits can be released after capture. Overall, these techniques have only been applied to mRNA sequencing; furthermore, because the Illumina sequencer only allows for 150–300 base-pair libraries, it is difficult to create libraries with the entire sequence of mRNAs. Instead, only 50 base pairs of the mRNA sequence from the poly A tail are used to identify and molecularly encode the expression levels. Therefore, drop-seq and hydrogel-based droplet sequencing are inappropriate for applications for analyzing splicing or mutations.

Droplet-based sequencing technology can be applied to DNAs. Abate's group reported single-cell genome sequencing with ultrahigh-throughput (SiC-seq) and microfluidic droplet barcoding.^39^ The bottleneck of droplet-based DNA sequencing is the labeling of all DNA fragments originating from the same cell. However, with Tn5 transposases that cut the DNA molecule at specific lengths and simultaneously insert the intended barcode, long DNA molecules can be fragmented into an adequate length with barcodes. Although the quality of sequencing results still requires improvement, this method has shown that DNA can be analyzed through droplet-based technologies. Currently, 10× Genomics is based on principles similar to those of the droplet-based single-cell separation methods mentioned above.^40,41^ Additionally, 10× Genomics provides information on single-cell gene expression, single-cell immune profile, genome sequence, exome sequence, and de novo assembly. This platform involves starting with either DNAs or cells as the input. Based on a Poisson distribution, the input is encapsulated within a droplet with gels containing barcodes. This platform has been applied in cancer research,^42^ genetic health,^41^ and agrigenomics.^43^

### C. Beyond single-cell separation: Rare-molecule compartmentalization

Although they have not been applied to NGS, some microchips using droplet technologies were developed to analyze circulating tumor DNAs (ctDNAs).^44,45^ Because biopsy is a painful but necessary process in diseases such as cancer, large expenditures in time and monetary and social costs are pressing problems for patients.^18^ In particular, tissue and needle biopsies are invasive and labor-intensive; thus, there is a demand for less invasive, more convenient, and accurate methods for diagnosing diseases. Liquid biopsy is the analysis of tumor materials obtained in a minimally invasive or noninvasive manner through the sampling of blood or other body fluids. ctDNA, which is known to be released from solid tumors to the blood stream, is a major and promising target for liquid biopsy because it can provide important genotypic information regarding the patients' tumor without invasive and expensive processes such as tissue or needle biopsy.^19,20^ However, there are challenges in using ctDNA as a genetic source for cancer diagnosis because when DNAs are extracted from the blood, ctDNAs typically exist in minute amounts among other DNAs originating from different cells in the blood stream, e.g., white blood cells or epithelial cells. Furthermore, cancer genomes are highly heterogeneous. If a mixture of ctDNA and the other DNAs extracted from whole blood is sequenced together, it is difficult to detect the important cancer-specific mutations unless sequencing is performed at a very high depth, which is expensive. As discussed above, several biochemistries can be paired with droplet technologies. By integrating existing biochemistry methods, or developing novel ones, droplet technologies may become advantageous, as compared to compartmentalizing small molecules, for increasing the limits of detection of rare mutations or aberrations.

Compared to microfluidic-based single-cell separation methods, these droplet-based approaches yield higher throughputs because the droplet-generating device can generate thousands of droplets in a short period (1–2 h). However, because the droplets contain lysis buffers and biochemical reactions occur as soon as the droplet is formed, it is difficult to observe whole cells after droplet formation. Although doublets can be excluded after imaging the cells for microfluidic-based single-cell separation platforms, they must be filtered using bioinformatics after NGS for droplet-based platforms.

## III. LASER-BASED PLATFORMS USING BIOCHIPS

Although microfluidic-based techniques offer a much higher throughput of single-cell isolation, the techniques cannot screen or select specific cells while preprocessing, which can be important when sequencing rare cells among a larger pool of different cells. Micromanipulation-based techniques are advantageous in selecting the desired cells; fewer cells of interest can be chosen through micromanipulation, but genetic materials can be sequenced to a higher depth (Fig. 2). Furthermore, the techniques introduced allow for staining on chip, thus providing additional information on protein and RNA expression, cell phenotype, etc.

On a biochip that can separate or enrich cells to be investigated, micromanipulation using a laser can be utilized to separate single CTCs for NGS. In 2013, Posadas' group reported high-purity prostate CTC isolation using a polymer nanofiber-embedded microchip for whole exome sequencing [Fig. 2(a)]. In-between the chip holders, a laser capture dissection polymer film and electrospun poly-lactic-co-glycolic acid (PLGA) nanofibers are consecutively coated on top of a glass substrate. PLGA nanofibers are modified with streptavidin such that a biotinylated antiepithelial cell adhesion molecule (EpCAM) antibody can be attached. Epithelial cell adhesion molecules are widely expressed on epithelial cell membranes. Because normal blood cells are not of epithelial origin, the antibody will only adhere to epithelial cancer cells, which are thought to be shed from the tumor mass and circulated. The PDMS (polydimethylsiloxane) chaotic mixer on top of the PLGA nanofibers serves to increase the probability of the CTCs reacting with the anti-EpCAM antibodies. After the enrichment of rare cells, the glass substrate is observed under a microscope to distinguish CTCs from the WBCs (white blood cells). If any single CTC is found, an infra-red laser is used to lower a polymer sticky finger that adheres to the nanofibers. Subsequently, a UV laser is used to burn the perimeter of the area of interest, thus allowing single CTC isolation with the adhered sticky finger. Subsequently, the single CTCs undergo the GenomePlex WGA 4 method, which is known as a PCR (polymerase chain reaction)-based whole genome amplification method. The amplified library is whole-exome sequenced using NGS.

Similarly, Kwon's group reported single cancer cell or CTC isolation using the laser-induced isolation of microstructures on an optomechanically transferrable biochip^46^ [Fig. 2(b)]. Instead of using polymers that are readily dissected using UV laser, this technology uses a near infra-red laser (1054 nm) on indium tin oxide (ITO)-coated glass substrates. ITO readily vaporizes when exposed to a pulsed near-infra-red laser. Epithelial CTCs are enriched by a biochip with anti-EpCAM antibody-coated microstructures. The biochip is held with chip holders and covered with a PDMS channel lid, similar to the previously discussed work by Posadas' group. After the CTCs are captured by the antibodies, the lid is removed from the chip. The cells captured on the chip are immunofluorescently stained to identify whether the captured cells are CTCs or white blood cells. After confirmation, a pulsed laser was applied to the microstructure to sort CTCs to a retrieving tube for whole-genome amplification. This technique was applied to a breast cancer patient's blood. The same applies to TrueRepertoire.^47^ When fluidic compartmentalization is performed by the surface tension of liquids around the microstructures, a single bacterium can be trapped in the liquid chamber. When a heterogeneous population of homogeneous colonies was formed, these single colonies were isolated by pulsed laser, producing a repertoire of immune-cell sequences to target a certain antigen.

A slightly different approach that can separate single cells using laser micromanipulation was reported by Takeuchi's group.^48,49^ This work utilizes indentations that can fit a single cell, similar to the C1 platform. When a laser is focused on a spot next to the cells, the entrapped single cell is released to the flow and can be isolated for single-cell analysis. Although not yet applied to NGS analysis, this platform is less prone to contamination than other open-chip-based micromanipulation techniques.

## IV. FUTURE BIOCHIPS FOR NGS APPLICATIONS

Although microfluidic and laser-based microchips have made breakthroughs in the application of NGS to real-world problems, the full potential of biochips in NGS is just beginning to be revealed. This is simply because biological systems are very complex, and therefore, biological samples exist in a wide range of scales. For instance, biological features such as nucleic acids and proteins can exist in very low amounts among a mostly homogeneous pool of molecules, while more complex systems like organs are composed of a heterogeneous population of cells. Because of these differences and unique features of biological systems, preparation methods that correspond to each biological sample must be developed for NGS to answer some of the most important biological or clinical questions or meet the increasing demand for simple-yet-powerful healthcare devices. Therefore, we envision that smart biochip platforms covering the whole scale of biological samples will contribute to NGS applications (Fig. 3). In order to develop such platforms, biochip researchers would require a deep and broad understanding of biological systems. In this section of this review, we will describe four examples of NGS applications that address relevant biological and/or clinical problems and provide insights into how biochip technologies can be applied to NGS.

Drug screening for personalized medicine is one example where preprocessing biochips can be applied for NGS [Fig. 3(a)]. Because of the growing importance of personalized medicine, it is important to develop platforms that can help examine the toxicity or efficacy of multiple drugs in parallel.^49–52^ Also, because combinatorial and sequential treatment has been found to be more effective than single-drug treatment in many cases, the need for multiplexity in testing these drugs is growing.^53^ Here, biochips' unique advantages in parallelization and miniaturization would allow the plexity that is needed for multiplex drug-screening. Conventional drug-screening assays concentrate on measuring cell survival at specific doses/concentrations of drugs, but these screening methods cannot be applied where the targets to be cured are important cells (e.g., neurons). Instead of concentrating on cell survival in drug screening, gene expression changes induced by the drugs can be examined to better cure irreplaceable cells. NGS can be used on these platforms to measure changes in gene expression profiles after treatment with the drug candidates. For example, Song *et al.* introduced massively parallel drug screening biochips that can measure the effects of sequential drug treatments.^54^ The platform is composed of a cell-biochip with multiple microwells seeded with cells. When assembled with drug-biochips, which were fabricated by assembling multiple drug-laden microparticles, the cells on the cell-chip are monitored for any drug-response. If the platform can be further engineered to enable cell retrieval, the gene expression can be measured in cells using NGS. Likewise, similar drug-screening biochips that can be used for NGS will bring innovations in screening drugs for neurodegenerative diseases and neuropathies.

One of the pathological features of the human defense system against cancer is tumor-infiltrating immune cells. These swarminglike immune cells are observed in tumor tissue and are thought to be related to improved clinical outcomes.^58^ Because gene expression can indicate the functional activity of different cells, single-cell analysis comparing gene expression profiles of the tumor cells and immune cells was reported.^30,55^ Likewise, modern cancer medicine has its fundamental basis in pathological observations that have been archived for a long time, and some of the most important biological and clinical questions lie in deciphering this pathological repertoire at the molecular level. Therefore, biochips that have advantages in processing through miniaturization and parallelization have significant contributions. For example, laser-based dissection or ablation techniques can be used along with immune-cell enriching methods because laser-based techniques have their strengths in retaining imaging information connected to the sequencing results [Fig. 3(b)]. Because the throughput of these techniques is much lower than those of microfluidics-based platforms, biochips that target single cells while retaining pathological information could have a high clinical impact in connecting the archived information to newly generated molecular information.

T cells and B cells are lymphocytes that play a crucial role in cellular and humoral immunity, respectively. The T cell receptors (TCRs) and B cell receptors (BCRs) that reside on the surfaces of these two types of cells are responsible for binding to antigen-presenting cells and foreign antigens, respectively. Therefore, the TCR and BCR diversity of these immune cells is an important factor in determining one's immunity. The diversity in the repertoire of TCRs and BCRs of each different immune cell comes from genetic rearrangements, which can be analyzed through sequencing the encoding mRNAs in the immune cells. Therefore, immune-cell sequencing has garnered significant interest as the repertoire diversity of the immune cells within a person accounts for the medical history of the person.^55,56^ However, as discussed in the cell atlasing section herein, these immune repertoires exist in heterogeneous populations within a pool, rendering it difficult to detect rare sequences among the mixed pool when NGS is used. Therefore, researchers adopted droplet-based single-cell analysis techniques to analyze immune cells. Azizi *et al.* used FACS to enrich immune cells before using droplet-based single-cell isolation to increase the resolution of the single-cell analysis data.^55^ In the future, if a droplet generator is integrated with an immune-cell-selecting module within a chip, immune-cell sequencing can be extended such that it could be implemented for clinical use [Fig. 3(c)]. Some examples of immune-cell sorting modules include microFACS[22] and antibody-based capture.^57^ For example, the Cyto-Mine platform developed by Sphere Fluidics was specifically automated and designed for single-cell analysis by integrating droplet fluidics and nanowells. Considering that there are various single-cell-sorting biochips, the potential for biochip-based technologies to revolutionize this field is significant.

Cell-free DNA analysis in biological samples is a powerful sampling tool for monitoring the corresponding biological systems. For example, circulating tumor cell-free DNA that exists in minute amounts in human blood samples can be used as a liquid biopsy for monitoring cancer relapse or metastasis for patients.^59^ Another example is environmental DNA that exists in ecosystems.^60^ Through sequencing environmental DNA, individual numbers of important species can be monitored. In both cases, the technical bottleneck in effectively analyzing cell-free DNA is the low concentration. Because cell-free DNA exists in low concentrations, analyzing ultrarare mutations or allele fractions from NGS alone can be challenging. Also, because of systematic NGS errors, measuring mutations or allele fractions accurately is another area for technical improvement. In this regard, biochips that can preconcentrate nucleic acids^61^ could be utilized for effective NGS library preparation. If the starting material can be enriched through a biochip, the accurate detection rate of rare mutations and allele fractions will increase. On the other hand, because the starting material is often fixed, especially for liquid biopsy applications, different strategies can be taken. For example, Yeom *et al.* reported barcode-free NGS error validation for ultrarare variant detection using a laser-based DNA retrieval system on a sequencing biochip.^62^ If similar platforms can be applied to the biological problems stated above, platforms utilizing biochips will have a significant impact on the field of cell-free DNA analysis [Fig. 3(d)].

The above are just a few specific examples to demonstrate the potential advantages of biochips used in NGS applications. When the scalability of these biochips can be aligned to the varying scales of biological systems, biochips will have the potential to bridge NGS technologies and solutions for many unmet real-world needs.

## V. CONCLUSIONS

Since Richard Feynman's speech “there's plenty of room at the bottom,” biochip technologies have undergone cutting-edge developments. Among the advantages of biochip technologies are miniaturization and parallelization, thus facilitating groundbreaking discoveries through the integration of NGS. We herein focused on biochip technologies that separate biomolecules and have been applied to NGS, single-cell sequencing, and liquid biopsy applications. We divided biochip technologies into two major categories: microfluidics- and laser-based. However, biochip technologies do not solely rely on these applications. We envision that the next step in the biochip field is to design smart biochips that can address different scientific and clinical questions. We provided four examples in Sec. IV of this paper, but scalable biochips can be used for almost all biological questions that remain unanswered. For example, as we are living in the era of super bacteria for which antibiotics are limited, it is important to correctly identify the bacteria in an infected patient. Sequencing genomic regions that can identify the bacteria will provide a strong tool for the early diagnosis of bacterial infections. However, in the early stages, bacteria exist in small populations, thus complicating the extraction of bacterial DNA from human cells in the bloodstream. Therefore, to cope with the bacterial intrusion, it is necessary to enrich the bacterial cells in the early stage and identify them using sequencing technologies. To enrich the bacterial cells, lab-on-a-chip devices will be beneficial because of their advantages in miniaturization. Therefore, from bacterial identification to environmental DNA profiling, addressing a wide range of different biological questions will be the next endeavor, to utilize the full potential of NGS. Some other examples of biochips that are being developed extensively include organ-on-chip devices, *in vitro* drug screening platforms, antimicrobial susceptibility tests,^63^ particle-based biochips,^64,65^ inertial flow biochips,^66–68^ and point-of-care devices.^69,70^ All these biochips are theoretically compatible with the NGS platform, if slightly modified to be suitable for biochemistry technologies, whether they are available or being developed. Such convergence of biochip and biochemistry technologies will first result in innovations in biological and clinical research. Then, in the near future, user-friendly biochip technologies will bridge the gap between the general public and precision medicine through increasing accessibility of the NGS technologies.

## References

[1] J. Eid , A. Fehr , J. Gray *et al.*, “ Real-time DNA sequencing from single polymerase molecules,” Science 323, 133–138 (2009). 10.1126/science.1162986 19023044

[2] M. L. Metzker , “ Sequencing technologies—The next generation,” Nat. Rev. Genet. 11, 31–46 (2010). 10.1038/nrg2626 19997069

[3] J. J. Kasianowicz , E. Brandin , D. Branton , and D. W. Deamer , “ Characterization of individual polynucleotide molecules using a membrane channel,” Proc. Natl. Acad. Sci. 93, 13770–13773 (1996). 10.1073/pnas.93.24.13770 8943010 PMC19421

[4] P. Bergveld , “ Development of an ion-sensitive solid-state device for neurophysiological measurements,” IEEE Trans. Biomed. Eng. BME-17, 70–71 (1970). 10.1109/TBME.1970.4502688 5441220

[5] D. R. Bentley , S. Balasubramanian , H. P. Swerdlow *et al.*, “ Accurate whole human genome sequencing using reversible terminator chemistry,” Nature 456, 53–59 (2008). 10.1038/nature07517 18987734 PMC2581791

[6] T. D. Harris , P. R. Buzby , H. Babcock *et al.*, “ Single-molecule DNA sequencing of a viral genome,” Science 320, 106–109 (2008). 10.1126/science.1150427 18388294

[7] J. Shendure , G. J. Porreca , N. B. Reppas *et al.*, “ Accurate multiplex polony sequencing of an evolved bacterial genome,” Science 309, 1728–1732 (2005). 10.1126/science.1117389 16081699

[8] E. R. Mardis , “ The impact of next-generation sequencing technology on genetics,” Trends Genet. 24, 133–141 (2008).10.1016/j.tig.2007.12.00718262675

[9] N. Navin , J. Kendall , J. Troge *et al.*, “ Tumour evolution inferred by single-cell sequencing,” Nature 472, 90–94 (2011). 10.1038/nature09807 21399628 PMC4504184

[10] E. Z. Macosko , A. Basu , R. Satija *et al.*, “ Highly parallel genome-wide expression profiling of individual cells using nanoliter droplets,” Cell 161, 1202–1214 (2015). 10.1016/j.cell.2015.05.002 26000488 PMC4481139

[11] C. Gawad , W. Koh , and S. R. Quake , “ Single-cell genome sequencing: Current state of the science,” Nat. Rev. Genet. 17, 175–188 (2016). 10.1038/nrg.2015.16 26806412

[12] L. R. Yates , S. Knappskog , D. Wedge *et al.*, “ Genomic evolution of breast cancer metastasis and relapse,” Cancer Cell 32, 169–184.e7 (2017). 10.1016/j.ccell.2017.07.005 28810143 PMC5559645

[13] R. M. Kumar , P. Cahan , A. K. Shalek *et al.*, “ Deconstructing transcriptional heterogeneity in pluripotent stem cells,” Nature 516, 56–61 (2014). 10.1038/nature13920 25471879 PMC4256722

[14] C. Alix-Panabières and K. Pantel , “ Clinical applications of circulating tumor cells and circulating tumor DNA as liquid biopsy,” Cancer Discovery 6, 479–491 (2016). 10.1158/2159-8290.CD-15-1483 26969689

[15] C. Alix-Panabières and K. Pantel , “ Challenges in circulating tumour cell research,” Nat. Rev. Cancer 14, 623 (2014). 10.1038/nrc3820 25154812

[16] S. Pandey , K. Shekhar , A. Regev , and A. F. Schier , “ Comprehensive identification and spatial mapping of Habenular neuronal types using single-cell RNA-Seq,” Curr. Biol. 28, 1052–1065 (2018). 10.1016/j.cub.2018.02.040 29576475 PMC6042852

[17] D. Ofengeim , N. Giagtzoglou , D. Huh *et al.*, “ Single-cell RNA sequencing: Unraveling the brain one cell at a time,” Trends Mol. Med. 23, 563–576 (2017). 10.1016/j.molmed.2017.04.006 28501348 PMC5531055

[18] L. R. Yates , M. Gerstung , S. Knappskog *et al.*, “ Subclonal diversification of primary breast cancer revealed by multiregion sequencing,” Nat. Med. 21, 751–759 (2015). 10.1038/nm.3886 26099045 PMC4500826

[19] L. A. Herzenberg , R. G. Sweet , and L. A. Herzenberg , “ Fluorescence-activated cell sorting,” Sci. Am. 234, 108–118 (1976). 10.1038/scientificamerican0376-108 1251180

[20] A. L. Givan , *Flow Cytometry First Principles* ( John Wiley & Sons, 2013).

[21] J. Krüger , K. Singh , A. O'Neill *et al.*, “ Development of a microfluidic device for fluorescence activated cell sorting,” J Micromech. Microeng. 12, 324 (2002). 10.1088/0960-1317/12/4/324

[22] R. Gao , C. Kim , E. Sei *et al.*, “ Nanogrid single-nucleus RNA sequencing reveals phenotypic diversity in breast cancer,” Nat. Commun. 8, 228 (2017). 10.1038/s41467-017-00244-w 28794488 PMC5550415

[23] H. C. Fan , J. Wang , A. Potanina , and S. R. Quake , “ Whole-genome molecular haplotyping of single cells,” Nat. Biotechnol. 29, 51–57 (2011). 10.1038/nbt.1739 21170043 PMC4098715

[24] C. Gawad , W. Koh , and S. R. Quake , “ Dissecting the clonal origins of childhood acute lymphoblastic leukemia by single-cell genomics,” Proc. Natl. Acad. Sci. U. S. A. 111, 17947–17952 (2014). 10.1073/pnas.1420822111 25425670 PMC4273416

[25] J. Easton , V. Gonzalez-Pena , D. Yergeau *et al.*, “ Genome-wide segregation of single nucleotide and structural variants into single cancer cells,” BMC Genomics 18, 906 (2017). 10.1186/s12864-017-4286-1 29178827 PMC5702214

[26] L. Binan , J. Mazzaferri , K. Choquet *et al.*, “ Live single-cell laser tag,” Nat. Commun. 7, 11636 (2016). 10.1038/ncomms11636 27198043 PMC4876456

[27] M. F. Fontana , G. L. de Melo , C. Anidi *et al.*, “ Macrophage colony stimulating factor derived from CD4+ T cells contributes to control of a blood-borne infection,” PLoS Pathog. 12, e1006046 (2016). 10.1371/journal.ppat.1006046 27923070 PMC5140069

[28] A. Zeisel , A. B. M. Manchado , S. Codeluppi *et al.*, “ Cell types in the mouse cortex and hippocampus revealed by single-cell RNA-seq,” Science 347, 1138–1142 (2015). 10.1126/science.aaa1934 25700174

[29] B. B. Lake , R. Ai , G. E. Kaeser *et al.*, “ Neuronal subtypes and diversity revealed by single-nucleus RNA sequencing of the human brain,” Science 352, 1586–1590 (2016). 10.1126/science.aaf1204 27339989 PMC5038589

[30] W. Chung , H. H. Eum , H.-O. Lee *et al.*, “ Single-cell RNA-seq enables comprehensive tumour and immune cell profiling in primary breast cancer,” Nat. Commun. 8, 15081 (2017). 10.1038/ncomms15081 28474673 PMC5424158

[31] P. Dalerba , T. Kalisky , D. Sahoo *et al.*, “ Single-cell dissection of transcriptional heterogeneity in human colon tumors,” Nat. Biotechnol. 29, 1120–1127 (2011). 10.1038/nbt.2038 22081019 PMC3237928

[32] J. C. H. Tsang , Y. Yu , S. Burke *et al.*, “ Single-cell transcriptomic reconstruction reveals cell cycle and multi-lineage differentiation defects in Bcl11a-deficient hematopoietic stem cells,” Genome Biol. 16, 178 (2015). 10.1186/s13059-015-0739-5 26387834 PMC4576406

[33] A. A. Pollen , T. J. Nowakowski , J. Chen *et al.*, “ Molecular identity of human outer radial glia during cortical development,” Cell 163, 55–67 (2015). 10.1016/j.cell.2015.09.004 26406371 PMC4583716

[34] G. La Manno , D. Gyllborg , S. Codeluppi *et al.*, “ Molecular diversity of midbrain development in mouse, human, and stem cells,” Cell 167, 566–580.e19 (2016). 10.1016/j.cell.2016.09.027 27716510 PMC5055122

[35] A. K. Shalek , R. Satija , J. Shuga *et al.*, “ Single-cell RNA-seq reveals dynamic paracrine control of cellular variation,” Nature 510, 363–369 (2014). 10.1038/nature13437 24919153 PMC4193940

[36] T. Thorsen , R. W. Roberts , F. H. Arnold , and S. R. Quake , “ Dynamic pattern formation in a vesicle-generating microfluidic device,” Phys. Rev. Lett. 86, 4163–4166 (2001). 10.1103/PhysRevLett.86.4163 11328121

[37] P. B. Umbanhowar , V. Prasad , and D. A. Weitz , “ Monodisperse emulsion generation via drop break off in a coflowing stream,” Langmuir 16, 347 (1999). 10.1021/la990101e

[38] A. M. Klein , L. Mazutis , I. Akartuna *et al.*, “ Droplet barcoding for single-cell transcriptomics applied to embryonic stem cells,” Cell 161, 1187–1201 (2015). 10.1016/j.cell.2015.04.044 26000487 PMC4441768

[39] F. Lan , B. Demaree , N. Ahmed , and A. R. Abate , “ Single-cell genome sequencing at ultra-high-throughput with microfluidic droplet barcoding,” Nat. Biotechnol. 35, 640–646 (2017). 10.1038/nbt.3880 28553940 PMC5531050

[40] J. O. Kitzman , “ Haplotypes drop by drop,” Nat. Biotechnol. 34, 296–298 (2016). 10.1038/nbt.3500 26963554

[41] G. X. Y. Zheng , B. T. Lau , M. Schnall-levin *et al.*, “ Haplotyping germline and cancer genomes with high-throughput linked-read sequencing,” Nat. Biotechnol. 34, 303–311 (2016). 10.1038/nbt.3432 26829319 PMC4786454

[42] S. Müller , G. Kohanbash , S. J. Liu *et al.*, “ Single-cell profiling of human gliomas reveals macrophage ontogeny as a basis for regional differences in macrophage activation in the tumor microenvironment,” Genome Biol. 18, 234 (2017). 10.1186/s13059-017-1362-4 29262845 PMC5738907

[43] G.-Q. Zhang , K.-W. Liu , Z. Li *et al.*, “ The Apostasia genome and the evolution of orchids,” Nature 549, 379–383 (2017). 10.1038/nature23897 28902843 PMC7416622

[44] L. Gorgannezhad , M. Umer , M. N. Islam *et al.*, “ Circulating tumor DNA and liquid biopsy: Opportunities, challenges, and recent advances in detection technologies,” Lab Chip 18, 1174–1196 (2018). 10.1039/C8LC00100F 29569666

[45] L. De Mattos-Arruda and C. Caldas , “ Cell-free circulating tumour DNA as a liquid biopsy in breast cancer,” Mol. Oncol. 10, 464–474 (2016). 10.1016/j.molonc.2015.12.001 26776681 PMC5528975

[46] S. Kim , A. C. Lee , H.-B. Lee *et al.*, “ PHLI-seq: Constructing and visualizing cancer genomic maps in 3D by phenotype-based high-throughput laser-aided isolation and sequencing,” Genome Biol. 19, 158 (2018). 10.1186/s13059-018-1543-9 30296938 PMC6176506

[47] J. Noh , O. Kim , Y. Jung *et al.*, “ High-throughput retrieval of physical DNA for NGS-identifiable clones in phage display library,” MAbs 11, 532 (2019); bioRxiv:370809. 10.1101/370809 30735467 PMC6512904

[48] W.-H. Tan and S. Takeuchi , “ A trap-and-release integrated microfluidic system for dynamic microarray applications,” Proc. Natl. Acad. Sci. U. S. A. 104, 1146–1151 (2007). 10.1073/pnas.0606625104 17227861 PMC1783141

[49] W.-H. Tan and S. Takeuchi , “ Dynamic microarray system with gentle retrieval mechanism for cell-encapsulating hydrogel beads,” Lab Chip 8, 259–266 (2008). 10.1039/B714573J 18231664

[50] A. S. Crystal , A. T. Shaw , L. V. Sequist *et al.*, “ Patient-derived models of acquired resistance can identify effective drug combinations for cancer,” Science 346, 1480–1486 (2014). 10.1126/science.1254721 25394791 PMC4388482

[51] M. Yu , A. Bardia , N. Aceto *et al.*, “ Ex vivo culture of circulating breast tumor cells for individualized testing of drug susceptibility,” Science 345, 216–220 (2014). 10.1126/science.1253533 25013076 PMC4358808

[52] A. H. H. Wong , H. Li , Y. Jia *et al.*, “ Drug screening of cancer cell lines and human primary tumors using droplet microfluidics,” Sci. Rep. 7, 9109 (2017). 10.1038/s41598-017-08831-z 28831060 PMC5567315

[53] F. Eduati , R. Utharala , D. Madhavan *et al.*, “ A microfluidics platform for combinatorial drug screening on cancer biopsies,” Nat. Commun. 9, 2434 (2018). 10.1038/s41467-018-04919-w 29934552 PMC6015045

[54] S. W. Song , S. D. Kim , D. Y. Oh *et al.*, “ One-step generation of a drug-releasing hydrogel microarray-on-a-chip for large-scale sequential drug combination screening,” Adv. Sci. 6, 1801380 (2018). 10.1002/advs.201801380 PMC6364496 30775230

[55] E. Azizi , A. J. Carr , G. Plitas *et al.*, “ Single-cell map of diverse immune phenotypes in the breast tumor microenvironment,” Cell 174, 1293–1308.e36 (2018). 10.1016/j.cell.2018.05.060 29961579 PMC6348010

[56] V. Greiff , E. Miho , U. Menzel , and S. T. Reddy , “ Bioinformatic and statistical analysis of adaptive immune repertoires,” Trends Immunol. 36, 738–749 (2015). 10.1016/j.it.2015.09.006 26508293

[57] S. Nagrath , L. V. Sequist , S. Maheswaran *et al.*, “ Isolation of rare circulating tumour cells in cancer patients by microchip technology,” Nature 450, 1235–1239 (2007). 10.1038/nature06385 18097410 PMC3090667

[58] Y. Man , A. Stojadinovic , J. Mason *et al.*, “ Tumor-infiltrating immune cells promoting tumor invasion and metastasis: Existing theories,” J. Cancer 4, 84–95 (2013). 10.7150/jca.5482 23386907 PMC3564249

[59] C. Bettegowda , M. Sausen , R. J. Leary *et al.*, “ Detection of circulating tumor DNA in early- and late-stage human malignancies,” Sci. Transl. Med. 6, 224ra24 (2014). 10.1126/scitranslmed.3007094 PMC4017867 24553385

[60] L. S. Epp , S. Kruse , N. J. Kath *et al.*, “ Temporal and spatial patterns of mitochondrial haplotype and species distributions in Siberian larches inferred from ancient environmental DNA and modeling,” Sci. Rep. 8, 17436 (2018). 10.1038/s41598-018-35550-w 30498238 PMC6265258

[61] H. Kim , J. Kim , E.-G. Kim *et al.*, “ Optofluidic in situ maskless lithography of charge selective nanoporous hydrogel for DNA preconcentration,” Biomicrofluidics 4, 043014 (2010). 10.1063/1.3516037 PMC3026036 21267091

[62] H. Yeom , Y. Lee , T. Ryu *et al.*, “ Barcode-free next-generation sequencing error validation for ultra-rare variant detection,” Nat. Commun. 10, 977 (2019). 10.1038/s41467-019-08941-4 30816127 PMC6395625

[63] J. Choi , Y.-G. Jung , J. Kim *et al.*, “ Rapid antibiotic susceptibility testing by tracking single cell growth in a microfluidic agarose channel system,” Lab Chip 13, 280–287 (2013). 10.1039/C2LC41055A 23172338

[64] Y. Song , Y. Jeong , T. Kwon *et al.*, “ Liquid-capped encoded microcapsules for multiplex assays,” Lab Chip 17, 429–437 (2017). 10.1039/C6LC01268J 27995235

[65] D. Lee , A. C. Lee , S. Han *et al.*, “ Hierarchical shape-by-shape assembly of microparticles for micrometer-scale viral delivery of two different genes,” Biomicrofluidics 12, 031102 (2018). 10.1063/1.5030597 29774082 PMC5935507

[66] E. Sollier , D. E. Go , J. Che *et al.*, “ Size-selective collection of circulating tumor cells using Vortex technology,” Lab Chip 14, 63–77 (2014). 10.1039/C3LC50689D 24061411

[67] S. L. Stott , C.-H. Hsu , D. I. Tsukrov *et al.*, “ Isolation of circulating tumor cells using a microvortex-generating herringbone-chip,” Proc. Natl. Acad. Sci. U. S. A. 107, 18392–18397 (2010). 10.1073/pnas.1012539107 20930119 PMC2972993

[68] E. Ozkumur , A. M. Shah , J. C. Ciciliano *et al.*, “ Inertial focusing for tumor antigen-dependent and -independent sorting of rare circulating tumor cells,” Sci. Transl. Med. 5, 179ra47 (2013). 10.1126/scitranslmed.3005616 PMC3760275 23552373

[69] G. Svedberg , Y. Jeong , H. Na *et al.*, “ Towards encoded particles for highly multiplexed colorimetric point of care autoantibody detection,” Lab Chip 17, 549–556 (2017). 10.1039/C6LC01358A 28102419

[70] D. Y. Oh , H. Na , S. W. Song *et al.*, “ ELIPatch, a thumbnail-size patch with immunospot array for multiplexed protein detection from human skin surface,” Biomicrofluidics 12, 031101 (2018). 10.1063/1.5032170 30867857 PMC6404946

